# The Dose-Dependent Effects of Fluorocitrate on the Metabolism and Activity of Astrocytes and Neurons

**DOI:** 10.3390/brainsci15020099

**Published:** 2025-01-21

**Authors:** Huiling Zhuang, Deliang Yuan, Fuxiu Shi, Xujun Wu, Zhen Luo, Wenbiao Gan

**Affiliations:** 1 School of Chemical Biology and Biotechnology, Peking University Shenzhen Graduate School, Shenzhen 518055, China; huiling_zhuang@pku.edu.cn (H.Z.); luozhenpku@163.com (Z.L.); 2 Institute of Neurological and Psychiatric Disorders, Shenzhen Bay Laboratory, Shenzhen 518132, China; yuandl@szbl.ac.cn (D.Y.); shifuxiu@szbl.ac.cn (F.S.); wuxj@szbl.ac.cn (X.W.)

**Keywords:** fluorocitrate, astrocyte metabolism, calcium signaling, neuronal calcium activity, synaptic plasticity

## Abstract

Background: Fluorocitrate (FC) ranging from 5 μM to 5 mM is often used as a specific metabolic inhibitor of the astrocytes to study astrocytic functions. Whether FC at such concentrations may affect neuronal metabolism and function *in vivo* remains unclear. Methods: We examined the effects of FC on the ATP levels and Ca^2+^ activity of the astrocytes and neurons in the motor cortices of living mice using two-photon microscopy. Results: We found that 25 μM and 250 μM of FC decreased the intracellular ATP levels and Ca^2+^ activity in the astrocytes in the motor cortex. Equally, 250 μM of FC, but not 25 μM of FC, reduced the intracellular ATP levels in the dendritic processes of the layer 5 pyramidal neurons. However, 25 μM of FC increased the neuronal Ca^2+^ activity, whereas ≥250 μM of FC decreased it. To test whether the differential effects of FC on neuronal Ca^2+^ activity reflect the direct effect of FC on the neurons or its indirect effect on the astrocytes, we used the CNO-hM3Dq chemogenetic approach to block astrocytic Ca^2+^ activity and examined the effect of FC. In the absence of astrocytic Ca^2+^ activity, 25 μM of FC still increased and ≥250 μM of FC reduced the dendritic Ca^2+^ activity of the neurons, respectively, suggesting a direct effect of 250 μM of FC on inhibiting neuronal Ca^2+^ activity. Further, 250 μM, but not 25 μM, of FC increased the size of the dendritic spines over 2 h. Conclusions: Our findings suggest that FC at high concentrations (≥250 μM) is not a specific inhibitor of astrocytic functions, as it directly affects neuronal metabolism and synaptic plasticity *in vivo*.

## 1. Introduction

Fluorocitrate (FC) inhibits the tricarboxylic acid (TCA) cycle by targeting aconitase [1,2], disrupting carbon flux, and impairing adenosine triphosphate (ATP) production [3,4,5]. Previous studies have shown that an intracerebral injection of FC temporarily inhibits the aconitase activity in the glial cells, causing citrate accumulation in the TCA cycle and subsequent disruption of glutamine synthesis [6]. FC also blocks astrocytic functions such as ATP production and Ca^2+^ signaling [4,5]. An infusion of 1 mM of FC into rat brains through a dialysis probe results in swelling of the glial cells over 4 h without affecting neuronal appearance [7]. As FC is absorbed preferentially by glial cells [8], it is often used as a specific metabolic inhibitor of astrocytes to study the effects of the astrocytes on neuronal functions [9,10,11,12,13,14,15].

FC at concentrations ranging from 5 μM to 5 mM has often been used to study glial cell functions in the brain [8,14,16,17,18,19,20,21,22,23,24]. For example, it has been shown that the inhibition of astrocytic function using 5 μM of FC alters endocannabinoid-dependent synaptic plasticity in the striatum [14]. Acute exposure to 100 μM of FC or 1 mM of FC reversibly depolarizes retrotrapezoid nucleus (RTN) astrocytes and increases the activity of the RTN neurons through a purinergic-dependent mechanism [18]. FC at concentrations of up to 5 mM has been used to inhibit astrocytic activity to study the involvement of astrocytes in synaptic transmission, including long-term potentiation (LTP) [23,24]. It is important to note that early studies suggested that specific effects of FC on astrocytic functions may occur within a narrow range [9]. For instance, while microinjections of 1 nmol of FC (1 μL) into rat striata have been shown to specifically affect the glial cells to alter the metabolism of the neurotransmitters, 2 nmol of FC (1 μL) causes irreversible degeneration of both the glia and neurons, potentially involving a direct effect of FC on the neurons [9]. Furthermore, the infusion of 1 mM of FC through a dialysis probe impairs glial functions in the cerebellum and decreases glutamine synthesis. Additionally, it also causes impairment of neuronal metabolic function, resulting in a sharp rise in glutamate, glycine, and GABA levels [7,25]. Thus, caution is warranted when perturbing astrocytic functions with FC, particularly at high concentrations, as the effect of FC treatment could reflect a direct impact on astrocytic or neuronal functions or both.

In this study, we investigated the dose-dependent effects of FC on astrocytic and neuronal metabolism and function in the cortices of living mice. We provide evidence that 25 μM of FC predominantly affects astrocytic functions, whereas 250 μM of FC not only affects the astrocytes but also reduces neuronal metabolism and functions *in vivo*. These findings suggest that FC at ~25 μM is advised for specific perturbation of astrocytic functions and that caution should be observed when interpreting data involving the use of ≥250 μM FC.

## 2. Materials and Methods

### 2.1. Animals

Thy1-YFP-H mice (Jackson Laboratory, Bar Harbor, ME, USA), expressing yellow fluorescent protein (YFP) primarily in the cortical layer 5 pyramidal cells, and C57BL/6J mice (Guangdong Provincial Medical Laboratory Animal Center, Guangzhou, China) were used in the experiment. All mice (both male and female, aged 4–5 weeks) were housed at the Peking University Shenzhen Graduate School in China under controlled conditions (22 ± 2 °C, a 12 h light/dark cycle) with unrestricted access to food and water. All of the experimental protocols were approved by the Institutional Animal Care and Use Committee of Shenzhen Graduate School, Peking University, and adhered to the institutional guidelines (protocol code: AP0011420; date of approval: 1 December 2019).

### 2.2. Drugs

FC (Sigma-Aldrich, St. Louis, MO, USA) was prepared following the procedures detailed in a previous study [9]. Briefly, 8 mg of barium FC salt was dissolved in 1 mL of 0.1 M HCl. Ba^2+^ was precipitated with 3 drops of 0.1 M Na_2_SO_4_. Then, 2 mL of 0.1 M Na_2_HPO_4_ was added. After centrifugation, the supernatant was diluted with 0.9% NaCl to 1 mM and the pH adjusted to 7.4. The prepared solution was stored at −20 °C. Prior to its usage, FC was diluted to the desired concentration using an artificial cerebrospinal fluid (ACSF) solution consisting of 119, 2.5, 1, 2.5, 1.3, 26.2, and 10 mM of NaCl, KCl, NaH_2_PO_4_, CaCl_2_, MgCl_2_, NaHCO_3_, and glucose, respectively, with an adjusted pH of 7.4.

### 2.3. Surgical Preparations for Two-Photon Imaging

Twenty-four hours prior to imaging, the mice underwent surgery to attach the head holders. The mice were deeply anesthetized with an intraperitoneal injection of ketamine (100 μg/g) and xylazine (10 μg/g). After shaving its head and making a midline scalp incision to expose the rat’s skull, the periosteum over the skull surface was removed. We used a pen to mark the skull region of the primary motor cortex [26]. A cyanoacrylate-based glue layer was applied, and a head holder was attached using dental acrylic cement. The mice recovered for one day after surgery to minimize the effects of anesthesia.

Before imaging, we created an open-skull cranial window over the designated area as previously described [27]. Briefly, a high-speed drill was used to carefully thin the skull under a dissecting microscope. A small craniotomy window (1 mm × 1 mm) was made in the thinned area and immediately covered with a glass coverslip to reduce the motion of the exposed brain, and one edge was kept open to allow drug penetration. The mice were acclimated to the two-photon imaging apparatus for ~10 min to reduce potential stress caused by the head restraint and awake imaging.

### 2.4. Ca^2+^ Imaging in the Layer 5 Neurons and Layer 1 Astrocytes Expressing GCaMP

The genetically encoded Ca²⁺ indicator GCaMP6s was employed to monitor the neuronal activity in the mice’s motor cortices. AAV1-CaMKII-GCaMP6s (~10 nL, 1.46 × 10^13^ v.g./mL, WZ Biosciences Inc., Shangdong, China) was injected into layer 5 (at a 520–650 μm depth) of the motor cortex in 3-week-old mice using a glass microelectrode, and imaging was performed 2 weeks post-injection. To image the astrocyte somata, 200 nL of virus (AAV9-GFAP-GcaMP7f; Titer: 1.02 × 10^13^ v.g./mL; OBiO Tech, Inc., Shanghai, China) was injected into the mouse cortices. Imaging was performed 3–4 weeks after the viral injection.

*In vivo* two-photon Ca^2+^ imaging was conducted at a 20–70 μm depth using a Fluoview 1000 system (920 nm, Olympus, Tokyo, Japan) equipped with a Ti:sapphire laser (MaiTai DeepSee, Spectra Physics, Milpitas, CA, USA). A 25× objective at ~1 Hz (N.A. 1.1, 3× digital zoom) and a ~15 mW laser power was used for the superficial cortical imaging. Imaging was performed during 3 trials of treadmill running before and after FC incubation (for 15, 30, 45, or 60 min).

### 2.5. ATP Imaging of the Neurons and Astrocytes

The ATP sensor ATeam1.03-nD/nA (“ATeam”) [28,29] was specifically expressed in either the neurons (AAV1-CaMKII-ATeam; titer: 3.50 × 10^13^ v.g./mL; 50 nL; Shanghai SunBio Biomedical Technology Co., Ltd., Shanghai, China) or astrocytes (AAV2/5-GFAP-ATeam 1.03; titer: 5.05 × 10^13^ v.g./mL; 200 nL; Shanghai SunBio Biomedical Technology Co., Ltd., Shanghai, China) in the mice’s motor cortices. ATP imaging of the layer 1 neuronal processes or astrocytic somata was performed over the 3 trials of treadmill running before and 15 min after the FC treatment. The relative intracellular ATP levels in the neuronal processes or astrocytic somata were measured during a period of 1 min pre-run, 2 min during a run, and 2 min post-run.

### 2.6. Imaging of the Dendritic Spine Dynamics

Dendritic spine imaging was conducted following established methods [30,31,32,33]. We obtained image stacks of the dendritic segments using a two-photon microscope immersed in ACSF or FC. High-magnification images for spine quantification were acquired using a 3× digital zoom. Two to three stacks of image planes (169 × 169 μm, 1024 × 1024 pixels for 25× objectives, a 2 μs pixel dwell time, and a 0.75 μm Z-step size) were collected within 60 µm of the pial surface, generating 3D dendritic data in the area of interest. For multiple imaging, the same area was relocated using low-magnification stacks (1× zoom) and vascular landmarks from thinned skull photographs.

### 2.7. Manipulating the Astrocyte Activity with CNO-DREADD

To manipulate the astrocytic activity, designer receptors exclusively activated by designer drug (DREADD)-hM3D(Gq) viruses (AAV9-GFAP-hM3Dq (Gq)-mCherry; titer: 6.02 × 10^13^ v.g./mL; 300 nL) and AAV9-GFAP-GCaMP7f (titer: 1.02 × 10^13^ v.g./mL; 200 nL) were injected into the same location in the motor cortex. Clozapine N-oxide (CNO; Sigma-Aldrich) was administered intraperitoneally (i.p.) to activate DREADD. CNO was dissolved in saline to a concentration of 0.5 mg/mL. Imaging was performed before and 20 min after CNO administration (0.25 mL/25 g of body weight) to allow DREADD to be activated. The efficacy of DREADD manipulation was assessed through Ca^2+^ imaging of the astrocytic somata before and 20, 30, 40, and 60 min after CNO administration. To characterize the activity of the astrocytes, the Ca^2+^ activity was evaluated during periods of quiet wakefulness and running.

The effect of FC on neuronal activity was examined after manipulating the astrocytic activity using the CNO-hM3Dq approach. AAV9-GFAP-hM3Dq (Gq)-mCherry (titer: 6.02 × 10^13^ v.g./mL; 300 nL) and AAV1-CaMKII-GCaMP6s (titer: 1.46 × 10^13^ v.g./mL; 300 nL) were injected into the mice’s motor cortices. After blocking the astrocytic Ca^2+^ activity with the CNO-hM3Dq approach, the motor cortices were incubated with FC, followed by Ca^2+^ imaging of the neuronal dendrites.

### 2.8. Analysis of Intracellular ATP Levels

Intracellular ATP levels were measured according to changes in the YFP/CFP fluorescence ratio of ATeam [28]. The intracellular ATP levels in the neuronal processes and astrocytic somata were examined using Image J version 1.53e (National Institutes of Health, Bethesda, MD, USA). The regions of interest (ROIs) were generally selected to quantify the astrocytic somata with distinct features within the field of view. Circular regions with a diameter of 40 μm were labeled as ROIs to quantify the neuronal processes.

Changes in the intracellular ATP levels were measured according to ΔR/R_0_ based on the measurements of ATeam (the YFP/CFP fluorescence ratio). ΔR/R_0_ represents (R − R_0_)/R_0_, where R_0_ denotes the average of the YFP/CFP fluorescence ratio of ATeam within 1 min of resting before FC treatment. The average value of the intracellular ATP levels during the resting state of the mice before FC dosing represents the intracellular ATP during resting. The minimum value of the YFP/CFP fluorescence ratio during running represents the intracellular ATP levels during running.

### 2.9. Analysis of Somatic and Dendritic Ca^2+^

Dendritic Ca^2+^ transients were indicated by changes in the GCaMP6 fluorescence (F) signal. The Ca^2+^ transients in the dendrites, both at rest and during running, were examined using Image J version 1.53e. The ROIs were generally selected to quantify dendrites with clear and distinct features within the field of view. Additionally, the value of the Ca^2+^ transient, defined as ΔF/F_0_, was derived from the GCaMP6 fluorescence measurements (without the vascular background). ΔF/F_0_ represents (F − F_0_)/F_0_, where F_0_ denotes the average of the 10% minimum fluorescence intensity within 1 min [34,35]. The Ca^2+^ activity from active dendrites was undetected in over 10% of the fluorescence traces owing to the low frequency of the Ca^2+^ transients in the dendrites, occurring only a few times per minute. The averaged peak activity was calculated by averaging the peak ΔF/F_0_ of each Ca^2+^ transient over 1 min. The integrated activity was determined as the average of the Ca^2+^ transients per 1 min.

### 2.10. Analysis of Dendritic Spine Size

Dendritic pine size was quantified using established methods [36,37]. After subtracting the background, the spine size was measured according to the ratio of the fluorescence intensity in the spine (the intensity of all of the pixels covering the spine in the best focal plane) to that of the adjacent dendritic shaft. The fluorescence intensity was calculated as follows:

The ratio of spine head diameter to adjacent dendritic shaft diameter = (Area (of spine) × Mean OD (of spine) − Area (of spine) × Mean OD (of background))/(Area (of spine) × Mean OD (of dendrite) − Area (of spine) × Mean OD (of background)). Here, “Area” represents the number of pixels in an oval enclosing the spine head, and “Mean OD” is the average brightness of these pixels.

The mean OD of both the background and the dendrite was calculated from measurements taken adjacent to each spine, averaged per dendrite segment. Spine size changes were assessed by comparing measurements of the same dendritic segment across imaging sessions.

### 2.11. Statistical Analyses

Data are expressed as the mean ± standard error of the mean. The Kolmogorov–Smirnov test was used to determine the normality of the data distribution. For normally distributed data, a two-tailed Student’s *t* test was applied to compare two groups, and non-parametric tests were used to test for differences between groups with a non-normal distribution. Non-parametric tests, including the independent-samples Mann-Whitney U test and the related-samples Wilcoxon signed-rank test, were used for non-normally distributed data. Statistical significance was set at *p* < 0.05 in all of the analyses. * indicates a statistically significant difference at *p* < 0.05. All of the statistical analyses were performed using GraphPad Prism version 8.3.0 (GraphPad Software, San Diego, CA) or SPSS Statistics for Windows, version 25 (IBM, Armonk, NY, USA).

## 3. Results

### 3.1. FC Causes a Reduction in Intracellular ATP in the Astrocytes

FC suppresses aconitase conversion in the TCA cycle, resulting in reduced ATP generation in the astrocytes [3,4,6,16]. To understand the impact of FC on the intracellular ATP levels in the astrocytes better, we used the genetically encoded ATP indicator protein ATeam to measure the changes in intracellular ATP in the astrocytes of the motor cortex after incubation with either 25 μM or 250 μM of FC.

**Figure 1:**
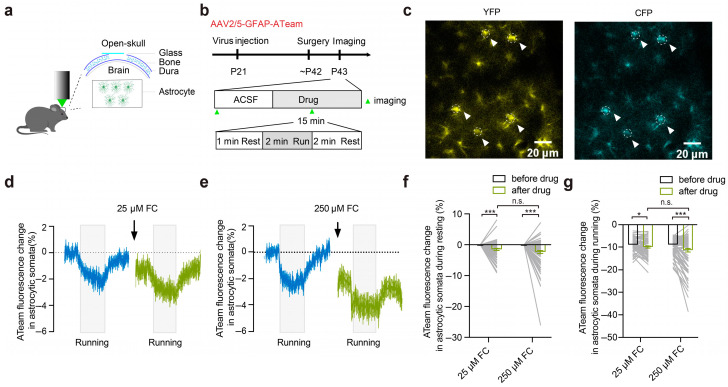
FC reduces the intracellular ATP levels in the astrocytes. (**a**,**b**) A schematic diagram of the experimental design. Injection of the AAV2/5-GFAP-ATeam virus into the motor cortex. Repeat imaging of astrocytic ATP was performed before and after the FC treatment. Green arrows indicate the time of imaging. (**c**) Expression of ATeam in the astrocytic somata (the white dotted line and white arrows indicate the selected ROI). (**d**,**e**) Decreased intracellular ATP levels in the astrocytes were induced by a mouse running. Resting: 0.00 ± 0.00%; running: −8.85 ± 0.34%; resting vs. running, *p* < 0.001. The intracellular ATP levels in the astrocytes in the motor cortex were decreased ~15 min after incubation with 25 μM (**d**) or 250 μM (**e**) of FC. The blue curve indicates the change in the astrocytic ATP levels during the ACSF treatment, the green curve represents the change in the astrocytic ATP levels 15 min after incubation with FC, and the gray box indicates that the mouse is running on a treadmill. (**f**) Statistical comparison of the intracellular ATP levels in the astrocytes before and after drug application during resting (25 μM of FC and 250 μM of FC). The 25 μM FC group: 0.00 ± 0.00% vs. −1.39 ± 0.32%, *p* < 0.001, 82 ROIs, 4 mice. The 250 μM FC group: 0.00 ± 0.00% vs. −2.22 ± 0.47%, *p* < 0.001, 92 ROIs, 3 mice. (**g**) The minimum intracellular ATP levels in the astrocytic somata during running were significantly reduced ~15 min after incubation with 25 μM and 250 μM of FC. The 25 μM FC group: −8.85 ± 0.34% vs. −9.86 ± 0.45%, *p* < 0.05, 74 ROIs, 4 mice. The 250 μM FC group: 8.83 ± 0.33% vs. −11.29 ± 0.78%, *p* < 0.001, 92 ROIs, 3 mice. * *p* < 0.05, *** *p* < 0.001. n.s., not significant.

We first performed imaging of ATeam in the astrocytes in the superficial layer of the motor cortex of head-restrained mice during quiet wakefulness and running (Figure 1a–c). The intracellular ATP levels in the astrocytes were significantly reduced during the 2 min period of motor running as compared to those during the quiet resting state, either before or after running (Figure 1d,e; resting: 0.00 ± 0.00%; running: −8.85 ± 0.34%; resting vs. running, *p* < 0.001).

During quiet wakefulness, the intracellular ATP in the astrocytes was significantly reduced after 15 min of incubation with 25 μM of FC (Figure 1d,f; 25 μM FC: 0.00 ± 0.00% vs. −1.39 ± 0.32%, *p* < 0.001; *n* = 82 ROIs from four mice) or with 250 μM of FC (Figure 1e,f; 250 μM FC: 0.00 ± 0.00% vs. −2.22 ± 0.47%, *p* < 0.001; *n* = 92 ROIs from three mice) as compared with that before FC incubation. Though not this was not significantly different, the 250 μM FC incubation tended to induce a larger reduction in ATP than 25 μM FC (Figure 1d–f; 25 μM FC: −1.39 ± 0.32%; 250 μM FC: −2.22 ± 0.47%, *p* = 0.38; *n* = 82 ROIs from four mice in the 25 μM FC group; *n* = 92 ROIs from three mice in the 250 μM FC group).

During 2 min of running, the intracellular ATP in the astrocytes was significantly lower after incubation with 25 μM of FC as compared to that before the drug treatment (Figure 1d,g; 25 μM FC: −8.85 ± 0.34% vs. −9.86 ± 0.45%, *p* < 0.05; *n* = 74 ROIs from four mice). In addition, 250 μM of FC also significantly reduced the intracellular ATP in the astrocytes during 2 min of running as compared to that before the drug treatment (Figure 1e,g; 250 μM FC: −8.83 ± 0.33% vs. −11.29 ± 0.78%, *p* < 0.001; *n* = 92 ROIs from three mice). No significant difference in the intracellular ATP levels was observed between the 25 μM FC and 250 μM FC treatment conditions (Figure 1d,e,g; 25 μM FC: −9.86 ± 0.45%; 250 μM FC: −11.29 ± 0.78%, *p* = 0.73; *n* = 74 ROIs from four mice in the 25 μM FC group; *n* = 92 ROIs from three mice in the 250 μM FC group).

Together, consistent with previous studies on FC-dependent inhibition of astrocytic metabolism [3,16], these results suggest that the treatment with 25 μM or 250 μM of FC causes a significant decrease in somatic intracellular ATP in the astrocytes of the motor cortex.

### 3.2. FC Reduces Astrocytic Ca^2+^ in the Motor Cortex

**Figure 2:**
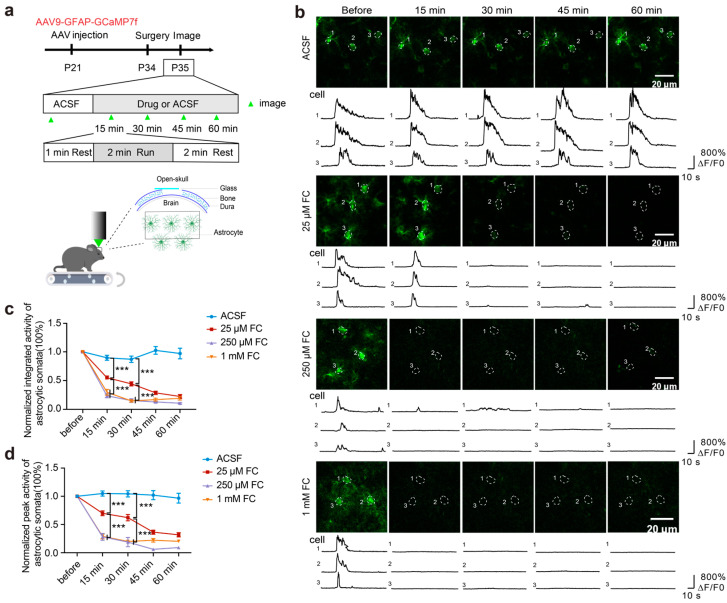
FC reduces astrocytic Ca^2+^. (**a**) Experimental paradigm. The AAV9-GFAP-GCaMP7f virus was injected into the motor cortex. Repeat imaging of the astrocyte Ca^2+^ activity was performed before and after FC or ACSF treatment. The green arrows indicate imaging. The Ca^2+^ activity of the astrocytes during the periods of 1 min pre-run, a 2 min run, and 2 min post-run was recorded (upper panel). (**b**) Images and fluorescent traces of representative astrocytic somata in the motor cortex expressing GCaMP7f in the ACSF- and FC-treated groups. The white circles indicate the somata. ΔF/F_0_ traces before and after the ACSF or FC treatment are presented at the bottom of the image panels. (**c**) Normalized integrated activity of the astrocytic somata after incubation with FC or ACSF. The normalized integrated astrocytic activity was significantly lower after the treatment with FC for ~15 min than after the ACSF treatment. ACSF: 0.90 ± 0.04; 25 μM FC: 0.55 ± 0.03; 250 μM FC: 0.24 ± 0.03; 1 mM FC: 0.30 ± 0.05; ACSF vs. 25 μM FC, *p* < 0.001; 25 μM FC vs. 25 μM FC, *p* < 0.001; 25 μM FC vs. 1 mM FC, *p* < 0.001. (**d**) Normalized peak activity of the astrocytic somata after incubation with FC or ACSF. Normalized peak activity of the astrocytic somata was significantly lower after the treatment with FC for ~15 min than after the ACSF treatment. ACSF: 1.049 ± 0.04; 25 μM FC: 0.70 ± 0.04; 250 μM FC: 0.28 ± 0.06; 1 mM FC: 0.29 ± 0.04; ACSF vs. 25 μM FC, *p* < 0.001; 25 μM FC vs. 250 μM FC, *p* < 0.001; 25 μM FC vs. 1 mM FC, *p* < 0.001. ACSF group: 82 somata from 6 mice; 25 μM FC group: 85 somata from 4 mice; 250 μM FC group: 69 somata from 5 mice; 1 mM FC group: 81 somata from 4 mice. *** *p* < 0.001.

FC-induced reductions in the intracellular ATP levels in the astrocytes may lead to the impairment of astrocytic functions *in vivo*. To test this possibility, we performed astrocytic Ca^2+^ imaging in the motor cortices of head-restrained mice subjected to treadmill running (Figure 2a). We found that the integrated activity and peak activity of the somatic Ca^2+^ transients in the astrocytes over the 2 min run were significantly reduced ~15 min after incubation with 25 μM of FC (Figure 2b–d. Integrated activity: ACSF = 0.90 ± 0.04; 25 μM FC = 0.55 ± 0.03; *p* < 0.001. Peak activity: ACSF = 1.049 ± 0.04; 25 μM FC = 0.70 ± 0.04; *p* < 0.001. *n* = 82 somata from six mice in the ACSF group. *n* = 85 somata from four mice in the 25 μM FC group.). The treatment with 250 μM of FC led to a further reduction in the integrated activity and peak activity of the somatic Ca^2+^ transients of the astrocytes as compared to that with 25 μM of FC (Figure 2b–d. Integrated activity: 25 μM FC = 0.55 ± 0.03; 250 μM FC = 0.24 ± 0.03; *p* < 0.001. Peak activity: 25 μM FC = 0.70 ± 0.04; 250 μM FC = 0.28 ± 0.06; *p* < 0.001. *n* = 85 somata from four mice in the 25 μM FC group. *n* = 69 somata from five mice in the 250 μM FC group.). In addition, when treated with 1 mM of FC, the astrocytic Ca^2+^ activity showed a larger reduction compared to that in the 25 μM FC-treated groups (Figure 2b–d. Integrated activity: 25 μM FC = 0.55 ± 0.03; 1 mM FC = 0.30 ± 0.05; *p* < 0.001. Peak activity: 25 μM FC = 0.70 ± 0.04; 1 mM FC = 0.29 ± 0.04; *p* < 0.001. *n* = 85 somata from four mice in the 25 μM FC group. *n* = 81 somata from four mice in the 1 mM FC group.). Furthermore, concentrations of 15 to 25 μM of FC also attenuated astrocyte calcium signaling, though the onset of this effect was delayed (Supplementary Figure S1a–c).

Consistent with previous studies of the effect of FC on astrocytic metabolism and functions [7,16,38,39,40,41,42], these results indicate that treatment with FC at various concentrations (25 μM to 1 mM) leads to significant decreases in the somatic Ca^2+^ activity in the astrocytes in the motor cortex.

### 3.3. FC Causes a Reduction in Intracellular ATP in the Neuronal Processes

**Figure 3:**
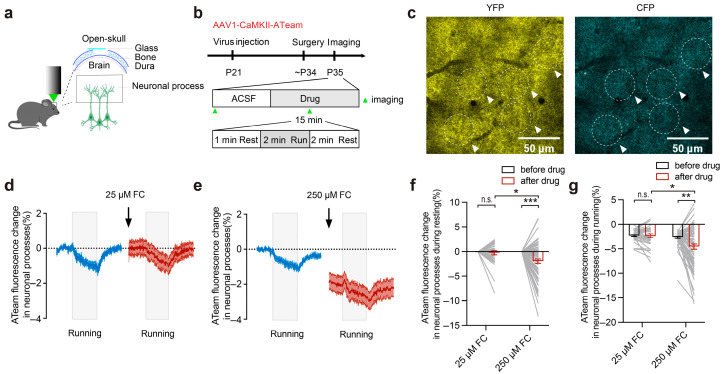
FC reduces intracellular ATP levels in the neurons. (**a**,**b**) A schematic diagram of the experimental design. Injection of the AAV1-CaMKII-ATeam virus into the motor cortex. Repeat imaging of ATP in the neuronal processes was performed before and after the FC treatment. Green arrows indicate the time of imaging. (**c**) The expression of ATeam in neuronal processes (the white dotted line and white arrows indicate the selected ROI). (**d**) Decreased intracellular ATP levels in the neurons are induced by the mouse running. Resting: 0.00 ± 0.00%; running: −2.33 ± 0.19%; resting vs. running, *p* < 0.001. The intracellular ATP levels in the neuronal processes were not significantly reduced after incubation with 25 μM of FC. (**e**) The intracellular ATP levels in the neuronal processes were significantly decreased after incubation with 250 μM of FC. The blue curve indicates the change in neuronal ATP levels during the ACSF treatment, the red curve represents the change in neuronal ATP levels 15 min after incubation with FC, and the gray box indicates that the mouse is running on a treadmill. (**f**) Statistical comparison of the intracellular ATP levels in the neuronal processes before and after drug application during resting (25 μM FC and 250 μM of FC). Black arrows indicate the starting point of imaging after 15 min of incubation with FC in the motor cortex. The 25 μM FC group: 0.00 ± 0.00% vs. −0.23 ± 0.42%, *p* = 0.89; 33 ROIs from 8 mice. The 250 μM FC group, 0.00 ± 0.00% vs. −1.96 ± 0.47%, *p* < 0.001; 70 ROIs from 14 mice. (**g**) Statistical comparison of the minimum intracellular ATP levels in the neuronal processes before and after drug application during running (25 μM of FC and 250 μM of FC). The 25 μM FC group: −2.33 ± 0.19% vs. −2.41 ± 0.36%, *p* = 0.94; 37 ROIs from 8 mice. The 250 μM FC group: −2.66 ± 0.18% vs. −4.56 ± 0.53%, *p* < 0.01; 70 ROIs from 14 mice. * *p* < 0.05, ** *p* < 0.01, *** *p* < 0.001. n.s., not significant.

Although FC is often used to specifically perturb astrocytic functions, several lines of evidence suggest that FC at high concentrations may also affect neuronal function [1,7,9,43,44,45]. To test this possibility *in vivo*, we investigated whether 25 μM or 250 μM of FC affected the intracellular ATP levels in the layer 5 pyramidal neurons in the motor cortex (Figure 3a–c). Prior to the FC treatment, we found that the intracellular ATP in the neuronal processes of the layer 5 pyramidal neurons was significantly reduced during the treadmill running period as compared to that during quiet wakefulness (Figure 3d; resting: 0.00 ± 0.00%; running: −2.33 ± 0.19%; resting vs. running, *p* < 0.001).

In the quiet resting state, we observed no significant decrease in the intracellular ATP levels in the neuronal processes of the layer 5 pyramidal neurons before and 15 min after incubation with 25 μM of FC (Figure 3d,f; 25 μM FC: 0.00 ± 0.00% vs. −0.23 ± 0.42%, *p* = 0.89; *n* = 33 ROIs from 8 mice). However, incubation with 250 μM of FC for 15 min significantly reduced the intracellular ATP levels in the neurons as compared to those before the FC treatment (Figure 3e,f; 250 μM FC: 0.00 ± 0.00% vs. −1.96 ± 0.47%, *p* < 0.001; *n* = 70 ROIs from 14 mice in the 250 μM FC group) or after the 25 μM FC treatment (Figure 3d–f; 25 μM FC: −0.23 ± 0.42%; 250 μM FC: −1.96 ± 0.47%; *p* < 0.5; *n* = 33 ROIs from 8 mice in the 25 μM FC group; *n* = 70 ROIs from 14 mice in the 250 μM FC group).

During the treadmill running period, we also observed no significant difference in the intracellular ATP levels before and after 15 min of the 25 μM FC treatment (Figure 3d,g; 25 μM FC: −2.33 ± 0.19% vs. −2.41 ± 0.36%, *p* = 0.94; *n* = 37 ROIs from 8 mice). In contrast, the 250 μM FC treatment for 15 min led to a significant decrease in the intracellular ATP levels during running compared to those before the drug treatment (Figure 3e,g; 250 μM FC: −2.66 ± 0.18% vs. −4.56 ± 0.53%, *p* < 0.01; *n* = 70 ROIs from 14 mice). Collectively, these results indicate that FC at 250 μM, but not at 25 μM, reduces the intracellular ATP levels in the neurons.

### 3.4. The Dose-Dependent Effects of FC on Dendritic Ca^2+^ Activity

**Figure 4:**
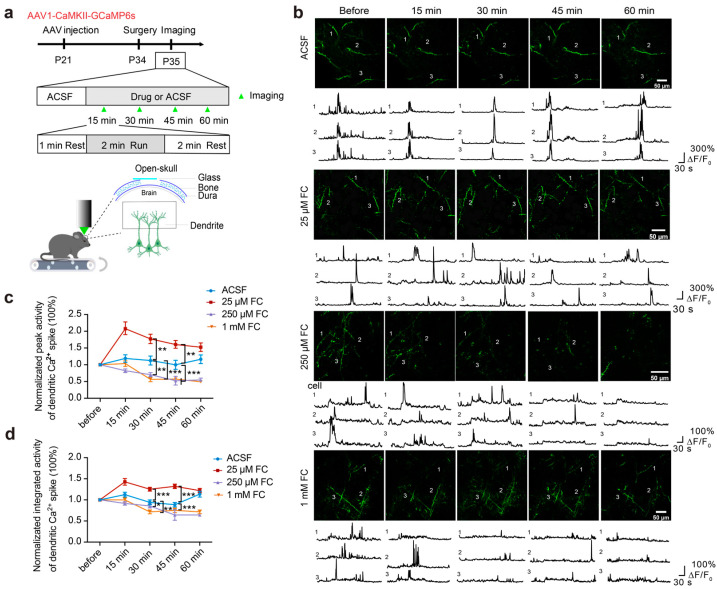
Dose-dependent effects of FC on the dendritic Ca^2+^ activity of the pyramidal neurons. (**a**) The experimental paradigm. The AAV1-CaMKII-GCaMP6s virus was injected into the motor cortex. Repeat imaging of the dendritic Ca^2+^ activity was performed before and after the treatment with FC or ACSF. The green arrowhead indicates the time period of the imaging (1 min pre-run, a 2 min run, and 2 min post-run). (**b**) Images and ΔF/F_0_ traces of representative dendrites of the pyramidal neurons expressing GCaMP6s in the ACSF- and FC-treated groups. (**c**) Normalized peak activity of dendritic Ca^2+^ during running was observed after incubation with FC or ACSF. Normalized peak activity after the treatment with 250 μM of FC and 1 mM of FC for ~30 min, but not 25 μM of FC, was significantly lower than that after the ACSF treatment. ACSF: 1.13 ± 0.13; 25 μM FC: 1.77 ± 0.14; 250 μM FC: 0.70 ± 0.06; 1 mM FC: 0.56 ± 0.06; ACSF vs. 25 μM FC, *p* < 0.01; ACSF vs. 250 μM FC, *p* < 0.01; ACSF vs. 1 mM FC, *p* < 0.001. (**d**) Normalized integrated activity during running was observed after incubation with FC or ACSF. Normalized integrated neuronal Ca^2+^ activity during running after the treatment with 250 μM of FC and 1 mM of FC for ~30 min, but not 25 μM of FC, was significantly lower than that before treatment or after ACSF treatment. ACSF: 0.94 ± 0.06; 25 μM FC: 1.25 ± 0.05; 250 μM FC: 0.86 ± 0.05; 1 mM FC: 0.72 ± 0.05; ACSF vs. 25 μM FC, *p* < 0.001; ACSF vs. 250 μM FC, *p* < 0.05; ACSF vs. 1 mM FC, *p* < 0.01. ACSF group: 103 dendrites from 3 mice; 25 μM FC group: 304 dendrites from 5 mice; 250 μM FC group: 225 dendrites from 5 mice; 1 mM FC group: 111 dendrites from 3 mice. * *p* < 0.05, ** *p* < 0.01, *** *p* < 0.001.

Because intracellular ATP in the neuronal processes of the layer 5 pyramidal cells was decreased by 250 μM of FC (Figure 3e–g), 250 μM FC treatment may also disrupt neuronal functions. To test this possibility, we performed Ca^2+^ imaging of the apical tuft dendrites of the layer 5 pyramidal neurons in the primary motor cortex before and 30 min after incubation with FC during running (Figure 4a).

Compared with the ACSF-treated controls, the peak and integrated activity of the dendritic Ca^2+^ transients increased 30 min after the 25 μM FC treatment (Figure 4b–d. Peak activity: ACSF = 1.13 ± 0.13; 25 μM FC = 1.77 ± 0.14; *p* < 0.01. Integrated activity: ACSF = 0.94 ± 0.06; 25 μM FC = 1.25 ± 0.05; *p* < 0.001. *n* = 103 dendrites from three mice in the ACSF group. *n* = 304 dendrites from five mice in the 25 μM FC group.). In contrast to the ACSF group, 250 μM and 1 mM of FC significantly reduced the normalized peak and integrated activity of the apical dendritic Ca^2+^ transients ~30 min after the FC treatment (Figure 4b–d. Peak activity: ACSF = 1.13 ± 0.13; 250 μM FC = 0.70 ± 0.06; 1 mM FC = 0.56 ± 0.06; ACSF vs. 250 μM FC, *p* < 0.01, ACSF vs. 1 mM FC, *p* < 0.001. Integrated activity: ACSF = 0.94 ± 0.06; 250 μM FC = 0.86 ± 0.05; 1 mM FC = 0.72 ± 0.05; ACSF vs. 250 μM FC, *p* < 0.05; ACSF vs. 1 mM FC, *p* < 0.01. *n* = 304 dendrites from five mice in the 25 μM FC group. *n* = 225 dendrites from five mice in the 250 μM FC group. *n* = 111 dendrites from three mice in the 1 mM FC group).

Together, these results reveal the dose-dependent effects of FC on the Ca^2+^ activity of the neuronal processes of the layer 5 pyramidal cells: FC at 25 μM increased the apical dendritic Ca^2+^ activity, whereas FC at 250 μM or 1 mM significantly decreased the apical dendritic Ca^2+^ activity.

### 3.5. High Concentrations of FC Reduce the Dendritic Ca^2+^ Activity in the Absence of Astrocytic Ca^2+^ Activity

Astrocytic Ca^2+^ activity is known to regulate neuronal functions [15,46,47,48], including neuronal Ca^2+^ activity [49,50,51,52]. Given that FC affects astrocytic metabolism and Ca^2+^ activity, it is possible that the dose-dependent effects of FC on the dendritic Ca^2+^ activity of the layer 5 pyramidal neurons may reflect a direct effect of FC on the neurons or an indirect effect of FC on the astrocytes or both. To distinguish these possibilities, we specifically reduced astrocytic functions using the Gq-coupled DREADD chemogenetic method and subsequently examined the effect of FC on neuronal Ca^2+^ activity.

**Figure 5:**
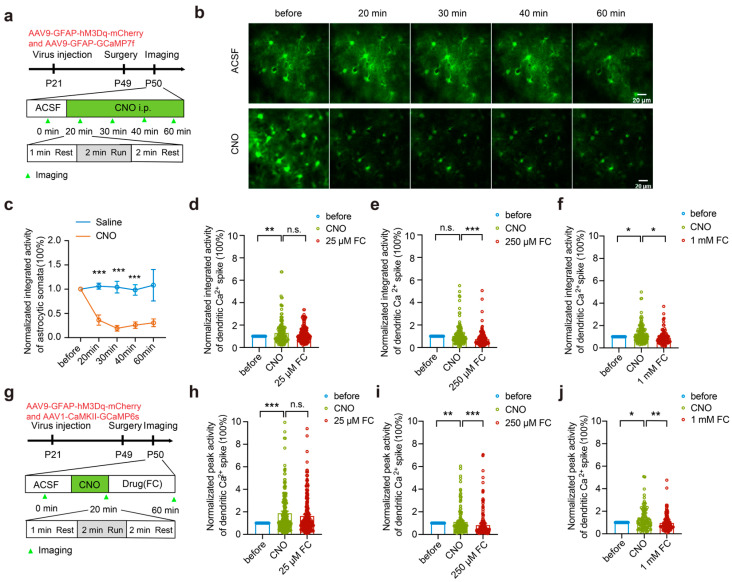
High concentrations of FC inhibit dendritic Ca^2+^ activity. (**a**) The experimental paradigm. Repeat imaging of astrocyte Ca^2+^ activity during running was performed before and after CNO injection. (**b**) Representative images of astrocytic Ca^2+^ activity in the motor cortex at different time points after ACSF (top) or CNO (bottom) administration. (**c**) Astrocytic Ca^2+^ activity was decreased 20–60 min after CNO administration. (**d**) Integrated dendritic Ca^2+^ activity is comparable before (ACSF treatment) and 30 min after incubation with 25 μM of FC. (**e**,**f**) The normalized integrated activity after 30 min of incubation with 250 μM ((**e**) CNO: 1.07 ± 0.07; 250 μM FC: 0.77 ± 0.05; *p* < 0.001) or 1 mM ((**f**) CNO: 1.22 ± 0.07; 1 mM FC: 0.94 ± 0.06; *p* < 0.05) of FC decreased significantly as compared to that before FC incubation. (**g**) The experimental paradigm. AAV9-GFAP-hM3Dq-mCherry and AAV1-CaMKII-GCaMP6s viruses were injected into the motor cortex to assess the impact of the FC treatment on dendritic Ca^2+^ activity after CNO injection. The 25 μM FC group: 170 dendrites from 3 mice. The 250 μM FC group: 165 dendrites from 3 mice. The 1 mM FC group: 98 dendrites from 3 mice. (**h**) The peak activity after 30 min of incubation with 25 μM of FC was not significantly different from that before incubation. Before: 1.00 ± 0.00; CNO: 1.89 ± 0.15; *p* < 0.001. (**i**,**j**) The peak activity after 30 min of incubation with 250 μM (CNO: 1.13 ± 0.10; 250 μM FC: 0.86 ± 0.08; *p* < 0.001) and 1 mM (CNO: 1.35 ± 0.08; 1 mM FC: 0.99 ± 0.08; *p* < 0.01) of FC was significantly decreased as compared to that before incubation. The 25 μM FC group: 281 dendrites from 3 mice; the 250 μM FC group: 165 dendrites from 3 mice; the 1 mM FC group: 102 dendrites from 3 mice. * *p* < 0.05, ** *p* < 0.01, *** *p* < 0.001. n.s., not significant.

We first measured the effect of hM3Dq activation using CNO in the astrocytes with Ca^2+^ imaging. hM3Dq and GCaMP7f were co-expressed in the astrocytes of the motor cortex (Figure 5a). After CNO-hM3Dq activation, the integrated activity of the astrocyte Ca^2+^ transients was significantly decreased (Figure 5b,c), indicating that the activation of hM3Dq by CNO resulted in a reduction in the Ca^2+^ activity in the astrocytes *in vivo*.

Next, we measured the effect of the FC treatment on the dendritic Ca^2+^ activity after reducing astrocytic Ca^2+^ using the CNO-hM3Dq approach. Twenty minutes after blocking the astrocytic Ca^2+^ activity, we found that the peak activity and integrated activity of the dendritic Ca^2+^ transients during running significantly increased (Figure 5d,h. Integrated activity: before = 1.00 ± 0.00; CNO = 1.33 ± 0.08; *p* < 0.01; *n* = 170 dendrites from three mice. Peak activity: before = 1.00 ± 0.00; CNO: 1.89 ± 0.15, *p* < 0.001; *n* = 282 dendrites from three mice.), indicating that the reduction in astrocytic Ca^2+^ activity led to an increase in dendritic Ca^2+^ activity. We then applied FC at doses of either 25 μM or 250 μM to the motor cortex and imaged the dendritic Ca^2+^ activity (Figure 5g). Notably, in the absence of astrocytic Ca^2+^, no significant difference in the peak activity and integrated activity of the dendritic Ca^2+^ transients was observed before and 30 min after the treatment with 25 μM of FC (Figure 5d,h. Integrated activity: CNO = 1.33 ± 0.08; 25 μM FC = 1.18 ± 0.05; *p* = 0.15; *n* = 170 dendrites from three mice. Peak activity: CNO = 1.89 ± 0.15; 25 μM FC = 1.65 ± 0.10; *p* = 0.18; *n* = 281 dendrites from three mice.). Together with the result of the small but insignificant effect of 25 μM of FC on neuronal ATP levels, this finding suggests that 25 μM of FC had no significant effect on the metabolism and activity of the layer 5 pyramidal neurons.

By contrast, when astrocytic Ca^2+^ was reduced using the CNO-hM3Dq approach, the peak and integrated activity of the dendritic Ca^2+^ transients during running significantly decreased ~30 min after the 250 μM FC treatment (Figure 5e,i. Integrated activity: CNO = 1.07 ± 0.07; 250 μM FC = 0.77 ± 0.05; *p* < 0.001; *n* = 165 dendrites from three mice. Peak activity: CNO = 1.13 ± 0.10; 250 μM FC = 0.86 ± 0.08; *p* < 0.001; *n* = 165 dendrites from three mice.). Similarly, a significant reduction in the peak and integrated activity of dendritic Ca^2+^ was also observed 30 min after the 1 mM FC treatment (Figure 5f,j. Integrated activity: CNO = 1.22 ± 0.07; 1 mM FC = 0.94 ± 0.06; *p* < 0.05; *n* = 98 dendrites from three mice. Peak activity: CNO = 1.35 ± 0.08; 1 mM FC = 0.99 ± 0.08; *p* < 0.01; *n* = 102 dendrites from three mice.). Thus, after blocking astrocytic Ca^2+^ using the CNO-hM3Dq approach, FC at concentrations of ≥250 μM reduced the dendritic Ca^2+^ activity, suggesting that high concentrations of FC likely have a direct inhibitory effect on dendritic Ca^2+^ activity.

### 3.6. High Concentrations of FC Increase the Size of the Dendritic Spines

FC at various concentrations (5 μM–1 mM) has been utilized to study the role of the effects of the astrocytes on synaptic plasticity [15,40,53,54]. Given our result that high concentrations of FC inhibit neuronal metabolism and functions, it is possible that previous findings on the impact of the astrocytes in regulating synaptic plasticity using a high concentration of FC may reflect, at least in part, a direct effect of FC on neuronal metabolism and activity. To explore the effect of FC on synaptic plasticity *in vivo*, we applied 25 μM or 250 μM of FC to the cortex and examined the impact of FC on dendritic spine size, a key indicator of synaptic strength [55,56,57].

**Figure 6:**
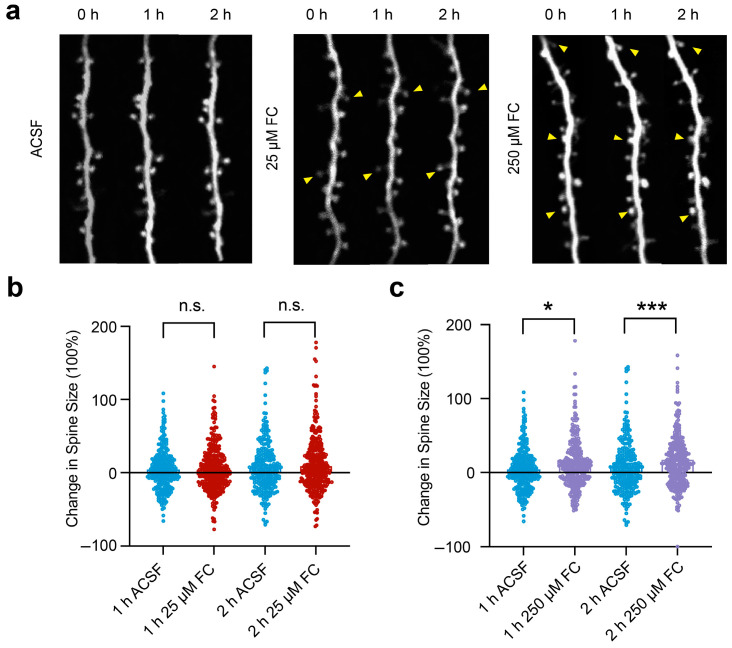
Astrocyte Ca^2+^ signaling was inhibited by FC, leading to changes in synaptic plasticity. (**a**) Schematic diagram of repeated imaging of dendritic spines before and after treatment with ACSF or FC (25 μM and 250 μM). Arrowheads indicate enlarged dendritic spines. (**b**) The change in dendritic spine size before and after ACSF or 25 μM FC treatment. No significant difference in spine size was observed before and after 25 μM FC treatment for 2 h. ACSF: 7.35 ± 2.16%; 25 μM FC: 10.12 ± 1.82%; *p* = 0.35. (**c**) Dendritic spine size was significantly larger after 250 μM FC treatment for 2 h than that in ACSF group. ACSF: 7.35 ± 2.16%; 250 μM FC: 18.29 ± 2.78%; *p* < 0.001. ACSF group (control group): 260 dendritic spines from 5 mice; 25 μM FC group: 388 spines from 3 mice; 250 μM FC group: 340 spines from 3 mice. * *p* < 0.05, *** *p* < 0.001. n.s., not significant.

Thy1-H line mice expressing YFP in their layer 5 pyramidal neurons were used to detect changes in the dendritic spine size before and after the FC treatment (Figure 6a). The treatment with a low concentration of FC (25 μM) for 2 h had no significant effect on dendritic spine size (Figure 6b; ACSF: 7.35 ± 2.16%; 25 μM FC: 10.12 ± 1.82%; *p* = 0.35; *n* = 260 spines from five mice in the ACSF group; *n* = 388 spines from three mice in the 25 μM FC group). Because 25 μM of FC reduced the astrocytic ATP and Ca^2+^ activity without affecting neuronal ATP, this result suggests that blockade of astrocytic function over 2 h does not significantly alter the dendritic spine size in the layer 5 pyramidal neurons in the motor cortices of mice under quiet resting conditions.

In contrast, the size of the spines of the apical dendrites in the layer 5 pyramidal neurons was significantly larger in the 250 μM FC-treated group (2 h incubation) than that in the control group (2 h incubation) (Figure 6c; ACSF: 7.35 ± 2.16%; 250 μM FC: 18.29 ± 2.78%; *p* < 0.001; *n* = 260 spines from five mice in the ACSF group; *n* = 340 spines from three mice in the 250 μM FC group). Given that structural changes in the dendritic spines are related to changes in synaptic strength [55,58,59], this finding suggests that FC at 250 μM increases synaptic strength. Because 250 μM of FC reduces the ATP in both the astrocytes and neurons, this finding further suggests that the effect of 250 μM of FC on increased spine size may reflect the direct effects of FC on the neurons.

## 4. Discussion

FC at various concentrations is commonly used as a specific inhibitor of astrocytic metabolism [1,5,13,16,44,60,61,62,63], but whether it also has a direct impact on neuronal metabolism and functions in the living brain remains unclear. Consistent with previous studies [3,16], our findings showed that 25 or 250 μM of FC caused significant decreases in the intracellular ATP levels in astrocytes of the primary motor cortex. On the other hand, 250 μM of FC, but not 25 μM of FC, significantly decreased the intracellular ATP levels in the neuronal processes of layer 5 pyramidal cells. Furthermore, after inactivation of astrocytic functions using a chemogenetic approach, we found that ≥250 μM of FC, but not 25 of μM FC, reduced the Ca^2+^ activity of the apical tuft dendrites of the layer 5 pyramidal neurons in the primary motor cortex. In addition, 250 μM of FC, but not 25 μM of FC, resulted in an increase in the dendritic spine sizes of the layer 5 pyramidal neurons. Taken together, these findings suggest that a low concentration of FC (<25 μM) primarily affects astrocytic metabolism, whereas a high concentration of FC (≥250 μM) has direct impacts on neuronal metabolism and activity.

Previous studies have used high concentrations of FC (≥250 μM) as a selective inhibitor of glial cells in order to study the functions of the glial cells in the nervous system [2,5,7,20,21,63,64,65,66,67]. For example, FC (1 mM) has been employed to study the involvement of astrocytes in nitric oxide production in the cerebella of awake rats [21]. Doses of 500 and 1000 μM of FC have been used to inhibit astrocytic metabolism, leading to a decrease in extracellular neurotransmitter glutamate levels but an increase in extracellular dopamine levels [63]. Additionally, it has been reported that FC (100–1000 μM) dose-dependently inhibits glial-mediated colonic contraction [64]. Our studies suggest that FC at <25 μM primarily affects astrocytic functions, but high concentrations of FC (≥250 μM) have effects on both the astrocytes and neurons. Therefore, caution is warranted in the data interpretation for experiments in which ≥250 μM of FC has been used to study the effects of inhibiting astrocytic metabolic activity on neuronal functions in the living cortex.

Our studies suggest that ≥250 μM of FC directly impairs ATP production in both the astrocytes and neurons. Intracellular ATP levels are crucial for neuronal functions such as neurotransmitter transmission and maintaining the homeostasis of potassium ions and Ca^2+^ [68,69,70]. Consistently, our data show that treatment with ≥250 μM of FC also reduces the Ca^2+^ activity in both astrocytes and neurons, which could be due to the reduced metabolism of the astrocytes and neurons. Additionally, neuronal Ca^2+^ activity plays a critical role in regulating various neuronal processes, such as excitability, neurotransmitter release, synaptic plasticity, associativity, and gene transcription [71,72,73]. Therefore, the reduction in ATP and Ca^2+^ activity with ≥250 μM of FC likely lead to the impairment of many neuronal functions in the brain.

High-concentration FC have been utilized to study the role of the astrocytes in regulating synaptic plasticity [24,74,75]. It has been shown that morphine treatment enhances long-term potentiation (LTP) and that the inhibition of the glial cells using FC (1 nmol/1 nL) blocks morphine-induced LTP enhancements [74]. Furthermore, FC (1 nmol/1 nL) treatment prevented high-frequency stimulus-induced LTP in the dorsal horn, which is dependent on glial activity [75]. Additionally, it has been reported that incubation with 5 mM of FC for more than 50 min blocks LTP at the excitatory hippocampal synapses in acute brain slices from adult rats [24]. These findings indicate that astrocytic activity plays a crucial role in the induction of LTP. However, given that high concentrations of FC may directly affect neuronal function, the conclusions drawn from the use of FC to inhibit the astrocytes and modulate synaptic plasticity need to be investigated further. Indeed, in mice in the resting state, our findings show that acute incubation with 25 μM of FC, which reduces astrocytic ATP and Ca^2+^, has no significant effects on dendritic spine size. On the other hand, 250 μM FC treatment, which reduces ATP and dendritic Ca^2+^ activity in both the astrocytes and neurons, leads to an increase in the size of the dendritic spines in layer 5 pyramidal neurons. These results raise the possibility that the effect of 250 μM of FC on increases in spine size may involve the reduced dendritic Ca^2+^ activity of the layer 5 pyramidal neurons and an activity-dependent homeostatic mechanism.

In summary, we provide evidence that 25 μM pf FC predominantly affects astrocytic functions, while ≥250 μM of FC not only affects the astrocytes but also directly reduces neuronal metabolism and functions *in vivo*. Caution is warranted in interpreting the results when FC is used at high concentrations to block astrocytic functions. Our results suggest the use of 15 to 25 μM of FC to investigate astrocytic function *in vivo*.

## 5. Conclusions

Although FC is often used to block metabolism in the astrocytes, FC at concentrations ≥250 μM leads to reduced neuronal metabolism and calcium activity and changes in synaptic plasticity in the living motor cortex. These findings suggest that it is essential to employ suitable concentrations of FC (15 to 25 μM of FC) to investigate the effects of astrocyte metabolism on the physiological functions of the neurons.

## Data Availability

The data presented in this study are available on request from the corresponding author. The data are not publicly available due to the sensitive information contained.

## Supplementary Materials

The following supporting information can be downloaded at https://www.mdpi.com/article/10.3390/brainsci15020099/s1. Figure S1: The effects of various concentrations of FC on astrocytic Ca^2+^. (a) The experimental paradigm. Repeat imaging of astrocyte Ca^2+^ activity was performed before and after different concentrations of FC. The green arrows indicate imaging. Ca^2+^ activity of the astrocytes during the periods of 1 min pre-run, a 2 min run, and 2 min post-run was recorded (upper panel). (b) Images and fluorescent traces of representative astrocytic somata in the motor cortex expressing GCaMP7f in groups treated with different concentrations of FC. ΔF/F_0_ traces before and after FC are presented on the right. (c) Normalized integrated activity of the astrocytic somata after incubation with different concentrations of FC.

## Abbreviations

The following abbreviations are used in this manuscript:

AAV	Adeno-associated virus
ACSF	Artificial cerebrospinal fluid
ATP	Adenosine triphosphate
CaMKII	Calcium/calmodulin-dependent protein kinase II
CNO	Clozapine N-oxide
DREADD	Designer receptors exclusively activated by designer drug
FC	Fluorocitrate
GCaMP	Genetically encoded calcium indicator
GFAP	Glial fibrillary acidic protein
hM3Dq	Human M3 muscarinic receptor DREADD
SPSS	Statistical Package for the Social Sciences
TCA	Tricarboxylic acid
YFP	Yellow fluorescent protein
