# Supplementary material for: The Dose-Dependent Effects of Fluorocitrate on the Metabolism and Activity of Astrocytes and Neurons
Source: Brain Sci. 2025 Jan 21;15(2):99. doi: 10.3390/brainsci15020099 (PMC11853058; doi:10.3390/brainsci15020099)
Supplement: Supplementary file 1 [file brainsci-15-00099-s001.zip › brainsci-3435010-supplementary.pdf]

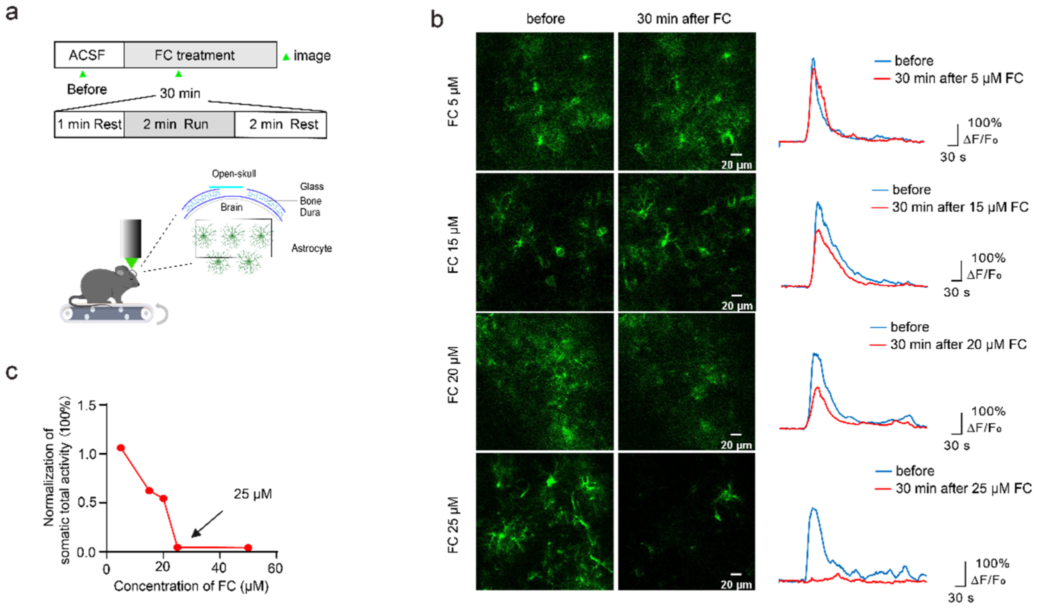

Supplement Figure S1. The effects of various concentrations of FC on astrocytic Ca^{2+}. (a) Experimental paradigm. Repeat imaging of astrocyte Ca^{2+} activity was performed before and after different concentrations of FC. The green arrows indicate imaging. Ca^{2+} activity of astrocytes during the period of 1 min pre-run, 2 min run, and 2 min post-run was recorded (upper panel). (b) Images and fluorescent traces of representative astrocytic somata in the motor cortex expressing GCaMP7f in groups treated with different concentrations of FC. $\Delta F/F_0$ traces before and after FC were presented on the right. (c) Normalized integrated activity of astrocytic somata after incubation with different concentrations of FC.
